# Nonlocal Response in Electrolytic Cells: A Generalized Poisson–Nernst–Planck Model with Memory Effects

**DOI:** 10.1021/acs.jpcb.5c06625

**Published:** 2025-12-21

**Authors:** Gabriel G. da Rocha, Michely P. Rosseto, Rodrigo J. Jaronski, Derik W. Gryczak, Luiz R. Evangelista, Rafael S. Zola, Ervin K. Lenzi

**Affiliations:** † Graduate Program in Science, 67883 State University of Ponta Grossa, Ponta Grossa 84030-900, PR, Brazil; ‡ Physics Department, 74354 Maringá State University, Maringá 87020-900, PR, Brazil; ¶ Physics Department, Federal University of Technology, Apucarana 86812-460, PR, Brazil

## Abstract

We present an extension of the standard Poisson–Nernst–Planck model by incorporating temporal memory effects to describe the spectroscopy impedance response in electrolytic systems. This model yields a modified current-density relation in which the ionic flux depends nonlocally on the applied electric field. The resulting electrical impedance may exhibit non-Debye relaxation and fractional-like scaling at low frequencies, providing a basis for anomalous diffusion in confined electrolytes. We analyze impedance spectroscopy data from NH_4_Cl-glycerol solutions for various concentrations to validate the model. The comparison demonstrates how the memory kernel governs the transition between normal and anomalous diffusion regimes, enabling accurate fits to experimental data. These results evidence the relevance of memory-driven transport in complex fluids and suggest a pathway to unify standard and fractional impedance models.

## Graphical abstract

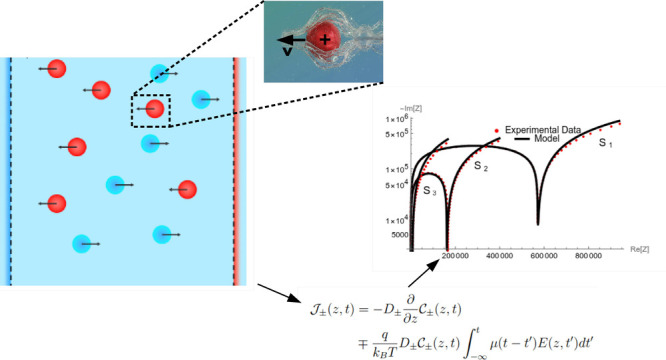

## 1 Introduction

Anomalous diffusion is a common phenomenon in complex systems.^1–3^ It deviates significantly from normal Brownian motion, which is characterized by Markovian processes and, consequently, by a linear time dependence for the mean-squared displacement. Anomalous diffusion exhibits a nonlinear time dependence of the mean-square displacement, thereby characterizing subdiffusion and superdiffusion. These behaviors have been observed in different contexts, and many approaches have been considered to analyze them.^4–8^ For example, subdiffusive behavior has been consistently observed in live cell imaging of proteins and organelles due to macromolecular crowding and viscoelasticity of the cytoplasm.^9,10^ Anomalous diffusion models describe the non-Gaussian transport of neurotransmitters and signaling molecules in dendritic spines and synaptic clefts.^11,12^ Fractional dynamics capture the aging and memory effects in complex fluids, polymer networks, and colloidal glasses.^13,14^ Environmental science also employs these models to describe the subdiffusive migration of contaminants in fractured rocks and soils, where traditional Fickian laws fail.^15,16^ Furthermore, in finance and human mobility, Lévy-like superdiffusion and heavy-tailed processes have been used to model asset price fluctuations and urban movement patterns.^17,18^ These applications are increasingly supported by models based on fractional partial differential equations,^19–22^ generalized Langevin equations,^23–25^ and continuous-time random walks (CTRWs),^26,27^ providing powerful tools to incorporate memory, heterogeneity, and nonlocality in both space and time. Such mathematical frameworks are essential to accurately describe the underlying dynamics observed in complex systems ranging from crowded intracellular environments,^26^ to ionic and subnuclear transport,^25^ to pollutant migration in heterogeneous media,^19^ and even to anomalous pattern recognition and classification in experimental data.^26^ Complex systems, such as confined electrolytes, viscoelastic media, biological membranes, polymer gels, and ionic liquids, often exhibit nonlocal behaviors over time, manifested by slow relaxation, hysteresis, and anomalous transport.^28–30^ On the other hand, the Poisson–Nernst–Planck (PNP) model gives a fundamental description of ionic transport in electrolytic media and is widely used in various contexts, including electrochemistry, cell biology, and materials physics.^23,31^ However, this model assumes that ionic fluxes respond instantaneously to applied electric fields and concentration gradients, implying a local relationship between force and flux. To capture such effects that are not suitably described in all frequency ranges, it is necessary to incorporate other mechanisms for the dynamics of ions, for example, by using time-fractional derivatives in continuity equations,^28,29^ generalized Langevin or fractional PNP formulations.^20,23,31^

Here, we analyze an extension of the Poisson–Nernst–Planck (PNP) model by modifying the drift term to include a nonlocal contribution, which can be related to the memory effects and noninstantaneous processes of relaxation associated with the electric field within the medium. To accomplish this task, we have structured our papers as follows. In Sec. 2, we extend the PNP model to incorporate anomalous behavior and in its framework we obtain closed expressions for the electrical impedance in the ac small–signal limit. In section 3, we compare the results of section 2 with the experimental data obtained for the electrolytic cells prepared by dissolving ammonium chloride (NH_4_Cl) in glycerol (Sigma-Aldrich). In section 4, we present our conclusions and discussions.

## 2 Extension of the PNP-Model

Before starting our discussion about the PNP-model, we consider the following scenario: the dynamics of an ion with charge *q* and velocity *v*(*t*), under the influence of a time-dependent electric field *E*(*t*), is described by the generalized Langevin equation:

1 $$m\frac{d}{dt}v\left(t\right)=-\int_{0}^{t}\Gamma \left(t-t^{\prime }\right)\;v\left(t^{\prime }\right)\;dt^{\prime }+qE\left(t\right)+\xi \left(t\right)$$

where *m* represents the mass of the ion under the action of an external electric field *E*(*t*), Γ­(*t*) is a kernel function representing the friction with memory effects and ξ­(*t*) is the random force with ⟨ξ­(*t*)⟩ = 0 and ⟨ξ­(*t*) ξ­(*t*′)⟩ = *k*_*B*_ *TΓ*(|*t* – *t*′|) . For this, we consider the ensemble average, which yields

2 $$m\frac{d}{dt}\left\langle v\left(t\right)\right\rangle =-\int_{0}^{t}\Gamma \left(t-t^{\prime }\right)\left\langle v\left(t^{\prime }\right)\right\rangle \;dt^{\prime }+qE\left(t\right)$$

This equation can be solved using the Laplace transform (L{v(t);s}=ṽ(s)) , which, in Laplace space, results in the following:

3 $$ṽ\left(s\right)=\frac{qẼ\left(s\right)}{\mathrm{ms}+\mathrm{\Gamma ̃}\left(s\right)}=q\mathrm{\mu ̃}\left(s\right)\;Ẽ\left(s\right)$$

with

4 $$\mathrm{\mu ̃}\left(s\right)=\frac{1}{\mathrm{ms}+\mathrm{\Gamma ̃}\left(s\right)},,\mathrm{where}\;\mu \left(t\right)=L^{-1}\left\{\mathrm{\mu ̃}\left(s\right);t\right\}$$

Applying the inverse Laplace transform:

5 $$\left\langle v\left(t\right)\right\rangle =q\int_{0}^{t}\mu \left(t-t^{\prime }\right)\;E\left(t^{\prime }\right)\;dt^{\prime }$$

From this equation, we can derive the current density associated with the motion of these ions, i.e., J(t)=C⟨v(t)⟩ , where C represents the ion concentration, which implies

6 $$J\left(t\right)=qC\int_{0}^{t}\mu \left(t-t^{\prime }\right)\;E\left(t^{\prime }\right)\;dt^{\prime }$$

Equation 6 shows that in the case of ions subjected to a medium whose dynamic aspect is governed by a generalized Langevin equation, the current density has a nonlocal dependence on the applied electric field, which, for the particular case μ­(*t*) ∝ δ­(*t*) (where δ­(*t*) is the Dirac’s delta) leads to obtaining J(t)∝qCE(t) . Thus, different choices of μ­(*t*) correspond to different friction kernels Γ­(*t*), capturing the properties of the medium such as viscoelasticity of the host medium and structural heterogeneity.

On the other hand, for the PNP model, we notice that the current density is given by

7 $$J_{\pm }^{\mathrm{PNP}}\left(z,t\right)=-D_{\pm }\frac{\partial }{\partial z}C_{\pm }\left(z,t\right)\mp \frac{q}{k_{B}T}D_{\pm }C_{\pm }\left(z,t\right)\;E\left(z,t\right)$$

where *D*_±_ is the diffusion coefficient related to the positive and negative ions. The first term corresponds to the diffusion of the ions, and the second term corresponds to the interaction of the ions with the electric field determined by the Poisson equation.

From eqs 6 and 7, one can observe that in the first case, the medium influences the current density through μ­(*t*), which defines the relaxation process. It can be connected to hydrodynamic effects with memory and correlated with stochastic noise inducing nonlocal temporal responses in the system.^32^ In the second case, the properties of the medium can be directly related to the diffusion coefficients of each ion in the bulk, and the term associated with the electric field is not influenced by the medium, as in the previous case, taking into account memory and hydrodynamic effects. These two models can be combined to result in the current density:

8 $$J_{\pm }\left(z,t\right)=-D_{\pm }\frac{\partial }{\partial z}C_{\pm }\left(z,t\right)\mp \frac{q}{k_{B}T}D_{\pm }C_{\pm }\left(z,t\right)\int_{-\infty }^{t}\mu \left(t-t^{\prime }\right)\;E\left(z,t^{\prime }\right)\;dt^{\prime }$$

which incorporates the effects of the eq 6 into eq 7. Note that we are using the limit of integrations from −∞ to *t* (following ref 33) since we are interested in the solution in a steady-state process as a result of a periodic applied potential (see below). The memory kernel introduces a delayed response that reflects the medium’s intrinsic microscopic features. This nonlocal coupling can be related to viscoelastic rearrangements, correlated thermal fluctuations, solvent reorganization, and heterogeneous local mobility. As a result, the ionic flux is no longer driven solely by the instantaneous electric field but by its entire weighted history, giving rise to non-Debye relaxation in the impedance and to subdiffusive transport in the low-frequency limit.

To obtain the linear response for a system subjected to a periodic external potential in this scenario, we consider eq 8 combined with the equations

9 $$\frac{\partial }{\partial t}C_{\pm }\left(z,t\right)+\frac{\partial }{\partial z}J_{\pm }\left(z,t\right)=0$$

and

10 $$\frac{\partial^{2}}{\partial z^{2}}\Phi \left(z,t\right)=-\frac{q}{\varepsilon }\left(C_{+}\left(z,t\right)-C_{-}\left(z,t\right)\right),,\mathrm{with}\;E\left(z,t\right)=-\frac{\partial }{\partial z}\Phi \left(z,t\right)$$

and appropriate boundary conditions. First, let us consider an electrolyte confined in a sample of thickness *d* separated by two electrodes located at *z* = −*d*/2 and *z* = *d*/2. The sample is subjected to an alternating electric field and the system is governed by eq 8, the continuity equation, eq 9, and Poisson’s equation, eq 10. Considering the ac small–signal limit, we have $C_{\pm }\left(z,t\right)=N+\eta_{\pm }\left(z\right)e^{i\omega t}$ , with $N\gg \left|\delta C_{\pm }\left(z,t\right)\right|$ (where $\delta C_{\pm }\left(z,t\right)=\eta_{\pm }\left(z\right)e^{i\omega t}$ ) and Φ­(*z*,*t*) = ϕ­(*z*)­e^i*ω t*^ (with Φ­(±*d*/2, *t*) = ± (*V*_0_/2)­e^i*ωt*^). For perfect blocking electrodes, i.e., $J_{\pm }\left(d/2,t\right)=J_{\pm }\left(-d/2,t\right)=0$ , it is possible to show that the electrical impedance is given by

11 $$Z\left(\omega \right)=\frac{2}{S\varepsilon i\omega \beta^{2}\left(i\omega \right)}\left\{\frac{\mu \left(i\omega \right)}{\lambda^{2}\beta (i\omega )\;}\;\tanh\left[\beta \left(i\omega \right)\frac{d}{2}\right]+\frac{d}{2D}i\omega \right\}$$

where S is the surface area of the electrode, μ­(i*ω*) is the kernel connected with the relaxation process of the system, where μ­(i*ω*) = ∫_0_^∞^ d*t*′ e^–i*ωt*′^μ­(*t*′), $\beta \left(i\omega \right)=\sqrt{\mu \left(i\omega \right)+\left(\lambda^{2}/D\right)i\omega }/\lambda$ , *D*_+_ = *D*_–_ = *D*, and $\lambda =\sqrt{\varepsilon k_{B}T/\left(2q^{2}N\right)}$ is the Debye length. Equation 11 recovers the standard case presented in ref 34 for the PNP model with perfect blocking electrodes for μ­(i*ω*) = 1, μ­(*t*) = δ­(*t*), and in the asymptotic limit of low frequency, we have

12 $$Z\left(\omega \right)\approx \frac{2}{S\varepsilon i\omega \beta^{2}\left(i\omega \right)}\left[\frac{\mu \left(i\omega \right)}{\lambda^{2}\beta \left(i\omega \right)}+\frac{d}{2D}i\omega \right]$$

With respect to μ­(i*ω*), it represents the physical process for ionic drift in a given medium. Hence, it is a phenomenological parameter chosen to represent the interaction between the ion and the medium. For example, for 1/μ­(i*ω*) ≈ (i*ωτ*)^γ^ (τ is a relaxation time) in the limit ω → 0 with 0 < γ < 1, we can approximate the previous result to

13 $$Z\left(\omega \right)\approx Z_{bulk}/{\left(i\omega \tau \right)}^{1-\gamma /2}$$

where $Z_{\mathrm{bulk}}=\tau /C_{\mathrm{bulk}}$ and *C*_*bulk*_ = *εS*/(2λ). This behavior has also been obtained in refs 35–37 by considering the fractional approach, i.e., the fractional time derivative in bulk or related to the surface effects. It should be noted that this behavior for the electrical impedance connected to the memory kernel μ­(*t*) exhibits features analogous to those of the constant phase elements (CPE) in the asymptotic limit of low frequency, commonly used in electrical spectroscopy impedance to account for distributed relaxation times and surface heterogeneities. Furthermore, according to the developments described in refs 38 and 39

14 ⟨(z−⟨z⟩)2⟩=π2ελ2∫0∞Re[σ(iω)]1−cos(ωt)ω2dω

which implies a connection between the electrical conductivity, σ­(ω), and the mean square displacement. This result implies that ⟨(*z* – ⟨*z*⟩)^2^⟩ ∼ *t* ^γ^ for Re­[σ­(i*ω*) ] ∼ ω^γ^ (0 < γ < 1); i.e., the underlying process is subdiffusive. It can be related to a random walk process asymptotically characterized by a long-tailed waiting time distribution. It is worth mentioning that Re­[σ­(i*ω*)] = constant implies ⟨(*z* – ⟨*z*⟩)^2^⟩ ∼ *t* (usual diffusion), which may be connected to a Poisson distribution for the waiting time. These arguments can be analyzed from the continuous time random walk approach, for example, by following the results presented in ref.,^38^ which allows us to connect the second moment with the mean square displacement for a separable probability density function,^4,6^ i.e., ψ­(*z*, *t*) = λ­(*z*) φ­(*t*), as follows: ⟨(*z* – ⟨*z*⟩)^2^⟩ ∝ φ̃(*s*)/[*s*(1 – φ̃(*s*))], where φ̃(*s*) is the waiting time distribution in the Laplace space. We can connect this result with the electrical conductivity, yielding: σ­(i*ω*) ∝ i*ωφ̃*(i*ω*)/[1 – φ̃(i*ω*)], which for a long-tailed distribution, i.e., φ̃(i*ω*) ∼ 1 – (i*ω*)^γ^, implies in Re­[σ­(i*ω*) ] ∼ ω^1−γ^ in the asymptotic limit of low frequency and, consequently, in a subdiffusion. For the case characterized by the eq 13, we obtain σ­(ω) ≈ σ_0_(*ωτ*)^1−γ/2^ with $\sigma_{0}=d\;\sin\left(\pi \gamma /2\right)/\left(SZ\right)$ , which allows us to show that ⟨(*z* – ⟨*z*⟩)^2^⟩ ∝ *t* ^γ/2^, where 0 < γ < 1, corresponding to a subdiffusion. This discussion demonstrates that the parameter γ associated with the kernel, beyond promoting the impedance connection with CPE elements, can capture a broad spectrum of time scales associated with structural heterogeneity and viscoelasticity, reflecting ion motion.

Figure 1 shows the behavior of the real and imaginary parts of the impedance when μ­(i*ω*) = *a* + (i*ωτ*)^γ–1^/[*a* + (i*ωτ*)^γ^] for different values of γ (0 < γ ≤ 1). We also show the standard PNP result, as indicated by the dashed black curve. For γ = 1.0, and *a* = 1 we obtain μ­(i*ω*) = 1 + (i*ωτ*)/[1 + (i*ωτ*)] and τ represents a relaxation time. This kernel represents a process with two contributions: one localized in time, described by the parameter *a*, and the second, which represents an exponential decay process (Debye relaxation) with decay time τ.^40^ This decay is associated with non-Markovian behavior in the drift process, characterized by a memory effect. This kind of kernel is particularly important in the low-frequency regime. We also observe that the real part of the impedance behaves similarly to what is observed for the PNP model in the presence of adsorption and desorption.^34^ The solid red curve is calculated for γ = 0.4 and *a* = 1, so μ­(i*ω*) = 1 + (i*ωτ*)^−0.6^/[1 + (i*ωτ*)^0.6^]. In this case, the kernel replaces exponential decay by a nonexponential decay process. In Figure 2, we present the Nyquist diagram and the electrical conductivity, using the same kernels as in Figure 1.

**Figure 1:**
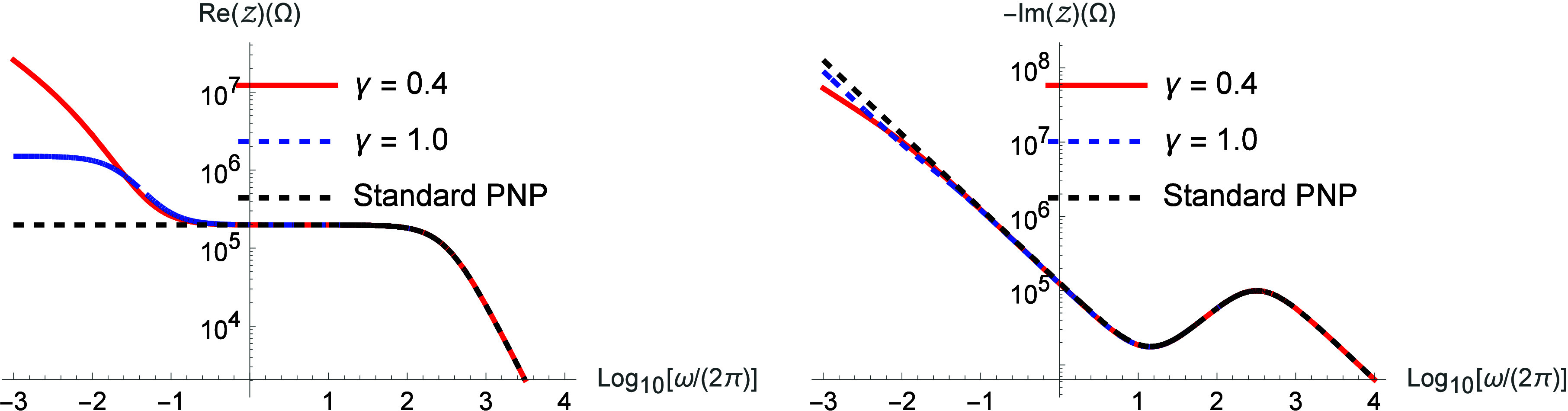
Real and imaginary parts of the impedance for different values of γ by considering μ­(i*ω*) = 1 + (i*ωτ*)^γ–1^/[1 + (i*ωτ*)^γ^]. We consider, for simplicity, *D* = 2 × 10^–9^ m/s^2^, τ = 10 s, *d* = 10^–3^ m, $S=\pi 10^{-3}{\;m}^{2}$ , *ε* = 90 *ε*_0_ (*ε*_0_ = 8.85 × 10^–12^ F/m), and λ = 9.97 × 10^–7^ m.

**Figure 2:**
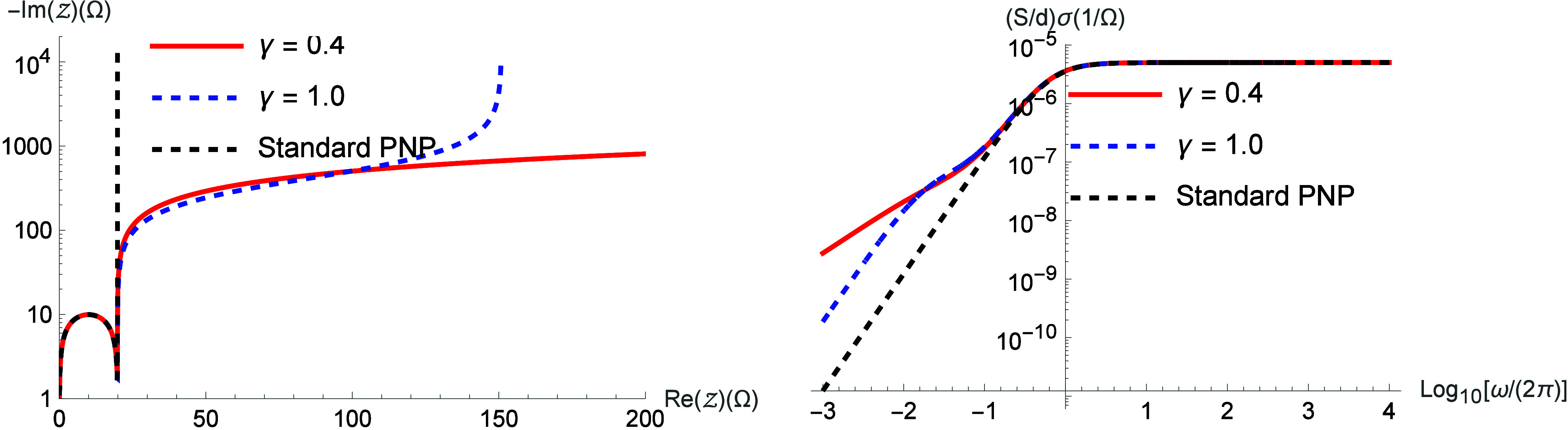
Nyquist diagram and electric conductivity for different values of γ by considering μ­(i*ω*) = 1 + (i*ωτ*)^γ–1^/[1 + (i*ωτ*)^γ^]. We consider, for simplicity, *D* = 2 × 10^–9^ m/s^2^, τ = 10 s, *d* = 10^–3^ m, $S=\pi 10^{-3}{\;m}^{2}$ , *ε* = 90 *ε*_0_ (*ε*_0_ = 8.85 × 10^–12^ F/m), and λ = 9.97 × 10^–7^ m.

Now, let us consider the boundary condition,

15 $$J_{\pm }\left(\pm d/2,t\right)=k\left[C_{\pm }\left(\pm d/2,t\right)-C_{eq}\right]$$

which corresponds to the Chang–Jaffe boundary condition.^41–44^ The Chang–Jaffe boundary condition, eq 15, is often used to represent specific ion adsorption.^45^ In eq 15, *k* represents the adsorption rate and $C_{\mathrm{eq}}$ is the concentration at thermodynamic equilibrium. In this case, it is possible to obtain the electrical impedance in the asymptotic limit of a small-ac and show that it is given by

16 $$Z\left(\omega \right)=\frac{2}{S\varepsilon i\omega \beta^{2}\left(i\omega \right)}\times \frac{(\mu \left(i\omega \right)/\lambda^{2}\beta \left(i\omega \right))\;\tanh\left(\beta \left(i\omega \right)d/2\right)+\left(d/\left(2D\right)\right)E\left(i\omega \right)}{1+(\beta \left(i\omega \right)k/\left(i\omega \right))\;\tanh\left(\beta \left(i\omega \right)d/2\right)}$$

with E(iω)=iω+kβ(iω)⁡tanh(β(iω)d/2) . Figure 3 illustrates the real and imaginary parts of the impedance. Figure 4 illustrates the Nyquist diagram and the electrical conductivity obtained from eq 16.

**Figure 3:**
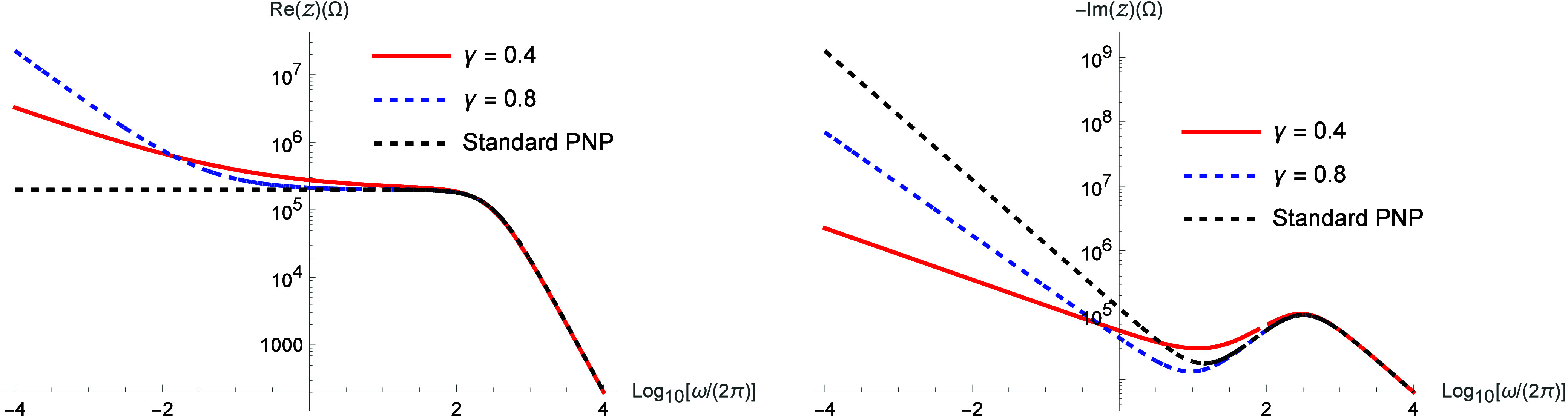
Real and imaginary parts of the impedance for different values of γ by considering μ­(i*ω*) = (i*ωτ*)^γ^/[1 + (i*ωτ*)^γ^]. We consider, for simplicity, *D* = 2 × 10^–9^ m/s^2^, τ = 10 s, *d* = 10^–3^ m, $S=\pi 10^{-3}{\;m}^{2}$ , *ε* = 90 *ε*_0_ (*ε*_0_ = 8.85 × 10^–12^ F/m), *k* = 1 m/s, and λ = 9.97 × 10^–7^ m.

**Figure 4:**
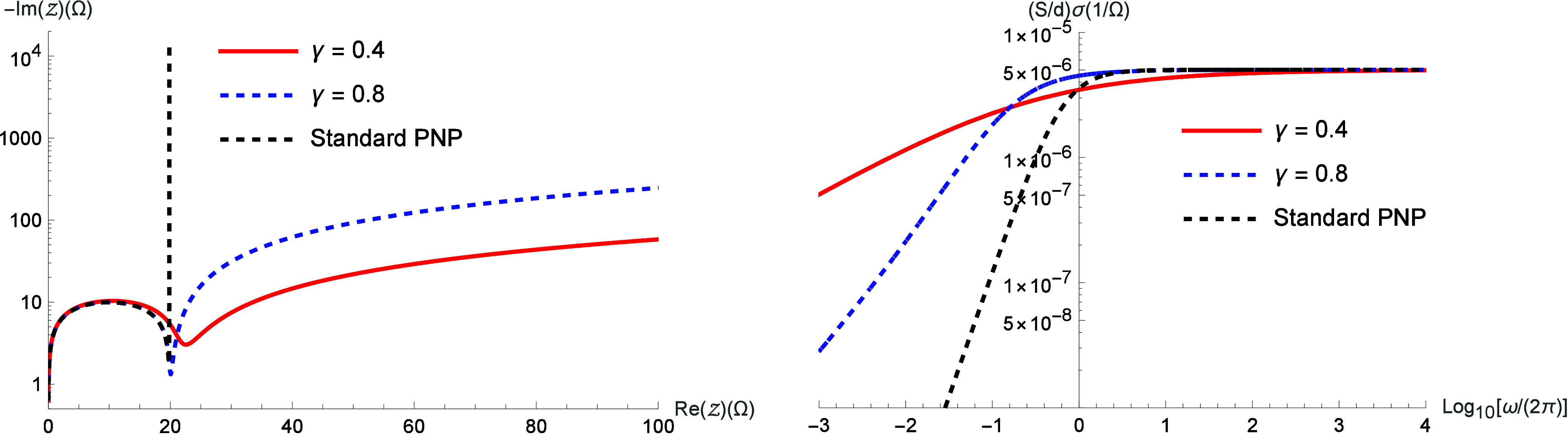
Nyquist diagram and electric conductivity for different values of γ by considering μ­(i*ω*) = (i*ωτ*)^γ^/[1 + (i*ωτ*)^γ^]. We consider, for simplicity, *D* = 2 × 10^–9^ m/s^2^, τ = 10 s, *d* = 10^–3^ m, $S=\pi 10^{-3}{\;m}^{2}$ , *ε* = 90 *ε*_0_ (*ε*_0_ = 8.85 × 10^–12^ F/m), *k* = 1 m/s, and λ = 9.97 × 10^–7^ m.

These figures show that the nonlocal effects introduced in the drift term have a significant influence on the system behavior in the asymptotic limit of low frequency, where the diffusion process has a pronounced impact. Similarly to the previous case, the behavior exhibited in Figures 3 and 4 can be attributed to anomalous diffusion.

## 3 Experimental Data and Impedance

We prepared samples by dissolving ammonium chloride (NH_4_Cl) in glycerol (Sigma-Aldrich) to compare our method with experimental data. Three different concentrations (all by weight %) were tested: (i) S_1_ was made with 0.0007% of NH_4_Cl; (ii) S_2_ containing 0.0048% of NH_4_Cl; and (iii) S_3_ with 0.1% of NH_4_Cl. Electrochemical impedance spectroscopy measurements were performed to compare the effect of the salt. Measurements were taken using a Hioki IM3533 LCR meter at room temperature, with a frequency range of 0.01 Hz to 200 kHz, and a small voltage of 25 mV. A circular sanded stainless steel electrode with a radius of 10 mm and a spacing of 1 mm between surfaces (see Figure 5a,b), was immersed in a container of 10 mL filled with the selected sample.

**Figure 5:**
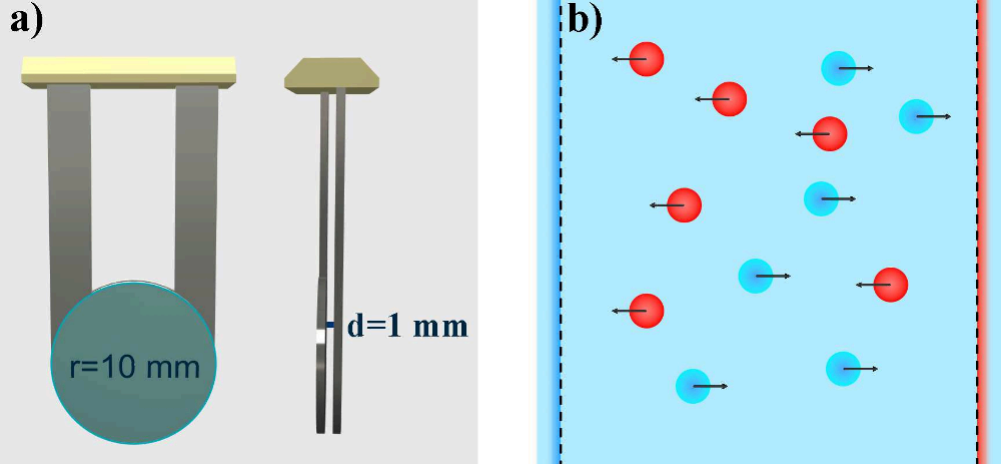
Panel a) shows a schematic representation of the circular steel electrode used in our experiments. Panel b) shows a schematic representation of electrophoretic ion migration under an alternating current (AC) field. Positively charged ions (cations, red spheres) migrate toward the negative electrode (cathode), while negatively charged ions (anions, blue spheres) migrate toward the positive electrode (anode). The polarity of the electrodes switches periodically with the applied AC, causing the ions to oscillate.

The dielectric behavior of different NH_4_Cl concentrations in glycerol was investigated using the extended PNP approach, presented in eq 16. The real part of the impedance is directly related to the system’s Ohmic resistance, which is influenced by the salt concentration, as evidenced by the plateau displacement observed in Figure 6. At lower frequencies, surface processes, adsorption, and desorption take place and are represented by parameters *k*. The diffusion behavior within the system can be analyzed by comparing the samples S_1_, S_2_, and S_3_. For S_1_, the parameters were *ε* ≈ 46 *ε*_0_, τ ≈ 10 s, γ ≈ 0.73, and λ ≈ 4.76 × 10^–7^ m, while for S_2_, *ε* ≈ 43*ε*_0_, τ = 4.0 s, γ = 0.66, and λ ≈ 2.34 × 10^–7^ m. For the highest concentration sample, S_3_, the parameters were *ε* ≈ 41*ε*_0_, τ ≈ 7.8 × 10^–2^ s, γ ≈ 0.75, and λ ≈ 4.8 × 10^–8^ m. These variations in λ reflect the salt concentration increases, which are inversely proportional to the number of charges. Also noteworthy is the fact that the dielectric constant decreases with the concentration of added salt. This dielectric decrement is directly related to the suppression of the collective response of the glycerol molecules.^46^ Note also that the experimental impedance data show that in glycerol-based solutions, the hydrogen-bonding network introduces delays in solvent reorganization, resulting in noninstantaneous transport.^32,47,48^ Figure 7 shows the electrical conductivity and Nyquist diagrams for the three NH_4_Cl concentrations. The theoretical model accurately reproduces the experimental behavior through the memory kernel μ­(i*ω*), which accounts for the distribution of relaxation times of glycerol-based electrolytes. This point is evident in the Nyquist representation, where the experimental data exhibit semicirculus and noncircular behaviors in the low-frequency, which is signature of non-Debye relaxation. These effects significantly influence the diffusion process and give rise to a memory effect in the ionic response. In addition, the viscosity and local mobility heterogeneities contribute to distributed relaxation times, which can be represented by power-law-type kernels.^7,49^ These features have implications for the kernel that must capture this behavior and provide a phenomenological explanation for the observed results.

**Figure 6:**
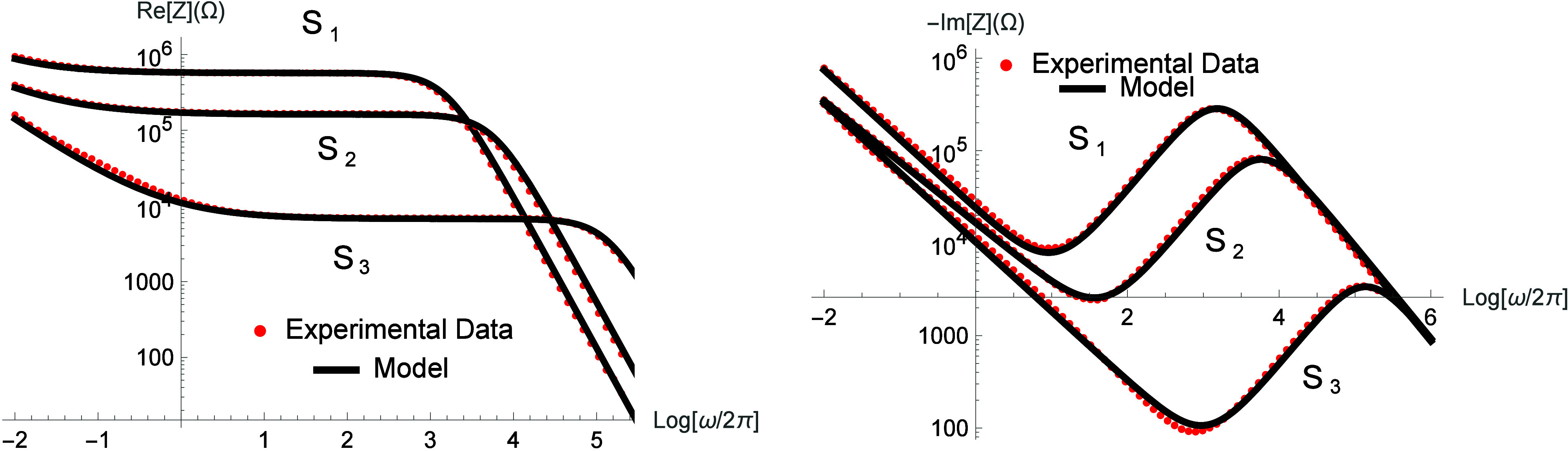
Real and imaginary parts of the impedance for different salt concentrations in glycerin. The red-dotted lines represent the experimental data, and the black solid lines represent the model used to describe the experimental data, with μ­(iω) = (iωτ)^γ^/[1 + (iωτ)^γ^]. For S_1_ the parameters used were *ε* ≈ 46*ε*_0_, τ ≈ 10 s, γ ≈ 0.73, and λ ≈ 4.76 × 10^–7^ m. For S_2_ the parameters used were *ε* ≈ 43*ε*_0_, τ = 4.0 s, γ = 0.66, and λ ≈ 2.34 × 10^–7^ m. For S_3_ the parameters used were *ε* ≈ 41 *ε*_0_, τ ≈ 7.8 × 10^–2^ s, γ ≈ 0.75, and λ ≈ 4.8 × 10^–8^ m. For these cases, *d* = 10^–3^ m, $S=\pi 10^{-3}{\;m}^{2}$ , *ε*_0_ = 8.85 × 10^–12^ F/m, *k* = 1.0 m/s, and *D* ≈ 2 × 10^–9^ m/s^2^.

**Figure 7:**
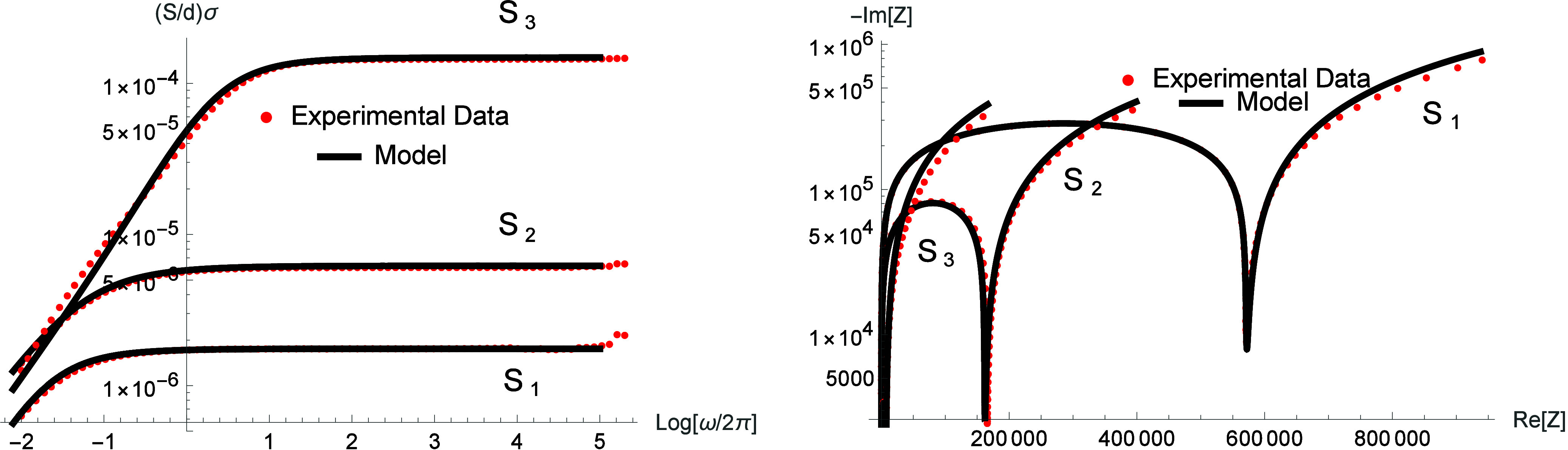
Electrical conductivity and Nyquist diagram for different salt concentrations in glycerin. The red-dotted lines represent the experimental data, and the black solid lines represent the model used to describe the experimental data, with μ­(iω) = (iωτ)^γ^/[1 + (iωτ)^γ^]. The parameter values are the same as Figure 6.

## 4 Discussions and Conclusions

We have extended the standard Poisson–Nernst–Planck (PNP) model to include temporal memory effects by means of a nonlocal relaxation kernel. By deriving a modified current-density relation from the generalized Langevin equation, we evidenced that ionic flux in electrolytic cells may depend nonlocally on the applied electric field, leading to a generalized impedance expression that accounts for non-Debye relaxation phenomena. The theoretical framework effectively reproduces the main features observed in experimental impedance spectroscopy (EIS) data obtained from the NH_4_Cl–glycerol solutions across a broad frequency range. Specifically, the model captures the transition between normal diffusion and anomalous subdiffusive regimes, as indicated by the scaling behavior of the memory kernel and the impedance response. This implies that memory-driven ionic transport may play an important role in confined electrolytic systems. Note also that the connection of the electrical conductivity with the mean square displacement and, consequently, with the waiting time distributions allows us to connect the diffusion of the ions with an anomalous scenario characterized by subdiffusion in the asymptotic limit of low frequency. The approach developed here bridges traditional diffusion models with more phenomenological descriptions that include nonlocal components, providing a rigorous yet flexible framework for understanding anomalous transport. While fractional differential operators are not directly employed in our formulation, the memory kernel can simulate fractional-like behaviors commonly associated with complex fluids.
